# The potential of Facebook advertising data for understanding flows of people from Ukraine to the European Union

**DOI:** 10.1140/epjds/s13688-022-00370-6

**Published:** 2022-12-06

**Authors:** Umberto Minora, Claudio Bosco, Stefano M. Iacus, Sara Grubanov-Boskovic, Francesco Sermi, Spyridon Spyratos

**Affiliations:** 1 Knowledge Centre on Migration and Demography, European Commission—Joint Research Centre, Via E. Fermi, 2749, I-21027 Ispra, (VA) Italy; 2 Institute for Quantitative Social Sciences, Harvard University, 1737 Cambridge St, K333, 02138 Cambridge, (MA) United States; 3 Global Relations and Cooperation Directorate, OECD Istanbul Centre, Asmalı Mescit, Meşrutiyet Cd. No:63, 34430, 02138 Beyoğlu/İstanbul, Turkey

**Keywords:** Ukraine, Armed conflict, Crisis response, Human migration, Innovative data, Facebook

## Abstract

This work contributes to the discussion on how innovative data can support a fast crisis response. We use operational data from Facebook to gain useful insights on where people fleeing Ukraine following the Russian invasion are likely to be displaced, focusing on the European Union. In this context, it is extremely important to anticipate where these people are moving so that local and national authorities can better manage challenges related to their reception and integration. By means of the audience estimates provided by Facebook advertising platform, we analyse the flows of people fleeing Ukraine towards the European Union. At the fifth week since the beginning of the war, our results indicate an increase in the number of Ukrainian stocks derived from Ukrainian-speaking Facebook user estimates in all the European Union (EU) countries, with Poland registering the highest percentage share (33%) of the overall increase, followed by Germany (17%), and Czechia (15%). We assess the reliability of prewar Facebook estimates by comparison with official statistics on the Ukrainian diaspora, finding a strong correlation between the two data sources (Pearson’s $r=0.9$, $p<0.0001$). We then compare our results with data on refugees in EU countries bordering Ukraine reported by the UNHCR, and we observe a similarity in their trend. In conclusion, we show how Facebook advertising data could offer timely insights on international mobility during crises, supporting initiatives aimed at providing humanitarian assistance to the displaced people, as well as local and national authorities to better manage their reception and integration.

## Introduction

Policies related to both disaster risk management and humanitarian assistance are designed with the aim of tackling the crises and associated challenges with preventive, preparedness, response and recovery actions. When it comes to population displacement as a consequence of conflicts, natural, or man-made disasters, there are specific challenges concerning populations’ health needs, safety, and well-being. In this context, availability of data is a crucial element as it can allow for rapid risk assessment and implementation of evidence-based risk management measures, yet there is still a need to improve the collection of disaster and conflict data ((EU)2021/836).^1^

In order to provide assistance in terms of provision of food, shelter, healthcare, and education to the displaced population, there is a need for timely and comparable data across countries. Recent events have shown that innovative data have the potential to integrate official data relevant for disasters and conflicts. The review of Bosco et al. [1] has shown that innovative data can offer a great geographic and temporal granularity, a (near-) real time availability, and an extensive coverage suitable for more immediate international comparisons.

In this context, the role of the Joint Research Centre (JRC) is (i) to support the EU Member States in the implementation of the Temporary Protection Directive for people fleeing Ukraine, and (ii) to assess the reliability of innovative data in support to EU policy-making for migratory crisis response.

Focusing on the current Russian military aggression against Ukraine, this paper aims to analyse the potential of innovative data for monitoring people fleeing Ukraine, and to contribute to the discussion on how such data can support crisis response. For this purpose, we monitor the flows of people fleeing Ukraine towards the EU using the weekly variation of Ukrainian-speaking Monthly Active Users (MAUs) estimates provided by Facebook’s advertising platform. These data represent the estimated number of people corresponding to a given set of user characteristics that have been active on Facebook’s family of applications in the past month (Sect. 4 describes these data in detail), and are not to be considered a proxy for monthly or daily active users on Meta.^2^ To see how these data are representative of the actual population, we compare them with official data on Ukrainian diaspora (*i.e.* stocks of Ukrainians living abroad) at national level in the EU countries provided by EUROSTAT and national statistical offices.

The paper is structured as follows: Sect. 2 offers a brief overview of the existing literature on innovative data for crisis response; Sect. 3 describes the context of the data needs of the Russian invasion of Ukraine; Sect. 4 presents the dataset used in this work and the methodology; Sect. 5 shows the findings of the analyses; finally, Sect. 6 discusses the results and concludes.

## Overview of the existing literature

In the context of crises requiring fast response, there is a growing body of scientific literature that draws on innovative data in order to estimate migration and population displacement as a consequence of natural disasters, man-made disasters and conflicts.

The first and most extensive usage of innovative data can be found in the literature studying the impact of natural disasters (floods, earthquakes, hurricanes, etc.) on human mobility and migration. *Call Detail Records (CDRs)* in specific, appear to be the most explored innovative data source for studying human mobility and migration induced by natural disasters. Several studies have employed CDRs to analyse mobility and migration caused by earthquakes in many different countries ([2–6]). For example, Lu et al. [3] analysed movements of mobile phone users from Haiti before and after the 2010 earthquake, Wilson et al. [4] provided detailed spatio-temporal estimates of population movements following the Gorkha earthquake in Nepal in 2015, while more recently the Flowminder Foundation [6] estimated population displacement caused by the 2021 Haiti earthquake. CDRs have been used to study the effect of other types of natural disasters on human mobility and migration as well. Isaacman et al. [7] used weather data and CDRs to model the impact on migration of severe drought in La Guajira, Colombia, in 2014. Also, Lu et al. [8] used CDRs to estimate the migration in Bangladesh in the short- (hours-week) but also in the long-term (years) period, following the 2013 Cyclone Mahasen. Overall, these studies highlight the potential of CDRs to estimate the effect of specific climate event on mobility and migration, also in small geographical areas and in short-time intervals.

Other types of innovative data have also been used for analysing the impact of natural disasters on migration and mobility, although less extensively than CDRs. For example, Rayer [9] used *Flight Passenger data* to estimate the effect of 2017 hurricanes on migration from Puerto Rico to Florida, while Jia et al. [10] used *Facebook displacement maps* to estimate population displacement during the Mendocino Complex and Woolsey fires in California. The latter showed that Facebook displacement maps can be used to estimate trends, magnitude, and spatial clustering of population displacement in case of disasters, although a representativeness bias remains in terms of demographic composition of the Facebook’s user base.

Over the past years there has been a rise in the number of research using innovative data to study epidemics and pandemics [11, 12]. The outbreak of the COVID-19 pandemic in particular has stimulated a lot of new “data for good” initiatives with the aim of supporting the risk assessment and identification of efficient risk management measures. Some examples of such data sharing initiatives include the release of mobility data to help the crisis response (e.g. *Apple Mobility Trends Reports*, *Google Community Mobility Reports*, *Baidu mobility data*). These data have been especially useful for gaining insights on the relationship between population mobility and the early spread of the SARS-COV2 virus [13–17].

Unlike natural disaster studies, the literature on conflict-induced migration has widely drawn on the so-called conflict and political violence event data, such as the Uppsala Con ict Data Program Georeferenced Event Dataset Global (UCDP), the Global Database of Events, Language, and Tone (GDELT), the Armed Conflict Location & Event Data Project (ACLED), the Global Terrorism Database (GTD), etc, collected using a semiautomatic annotation of events that appear in the news. Although these are not mobility data, this type of innovative data is frequently integrated with other type of innovative or traditional data to provide insights on conflict-induced migration. Carammia et al. [18], for example, integrated operational Google trends data with the Global Database of Events, Language, and Tone data to forecast the number of asylum applications in European countries for the coming four weeks. Suleimenova et al. [19] integrated Bing Maps, and data from United Nations High Commissioner for Refugees (UNHCR) with the Armed Conflict Location ; Event Data Project database to simulate refugee movements following conflicts in Burundi, Mali and Central African Republic.

Other types of innovative data for conflict and migration studies appear to be relatively less extensively employed. In this field, an important study of Corbane et al. [20] on the effects of the Syrian conflict showed that also open-access geospatial data (*Night-time satellite data* and JRC’s *Global Human Settlement Layer (GHSL)*) can be used to produce accurate and timely estimates on migration and mobility in conflict areas. Bharti et al. [21] combined night-time lights satellite imagery and anonymized mobile phone CDRs to analyse the population displacement in the context of the internal political conflict in Côte d’Ivoire in 2010. Similarly, with the crowdsourcing approach, the relevant information can be mined and used to analyse migration pathways following conflicts, however with significant methodological challenges [22].

Social media is another potential innovative data source for studying migration and conflicts. For example, geocoded Twitter data were used to infer estimates of internal mobility patterns [23], as well as emigration flows [24], following the migration crisis in Venezuela. In the same context, Palotti et al. [25] showed that also Facebook advertising platform can be used to assess in real time and at sub-national level the number of migrants and their socio-economic profiles. This paper aims to contribute to the latter strand of literature and assess how social media data, and in particular Facebook advertising data, can be used to monitor human mobility and migration during conflicts.

## The case study of Ukraine: context and data needs

As the Russian military aggression against Ukraine continues, the number of people forced to leave their houses relentlessly increases. In this war scenario, counting people who moves within the country, namely Internally Displaced Persons (IDPs), and those who have left the country in search of international protection (asylum seekers) becomes extremely difficult.

On the 16th of March 2022, there were almost 6.5 million people displaced in Ukraine as a direct result of the war, according to the Protection Cluster,^3^ a joint study by the UNHCR, the International Organisation on Migration (IOM), the United Nations Office for the Coordination of Humanitarian Affairs (UN-OCHA), and the Informing More Effective Humanitarian Action (REACH) published on the 18th of March.

The UNHCR provides a daily update of the estimated number of people who have fled Ukraine towards the neighboring countries after the military invasion.^4^ According to this source, on the 2th of April the number of people who have left the country was almost 4.2 million: 54% to Poland, 14% to Romania, 9% to Moldova, 9% to Hungary, 8% to Russia, 7% to Slovakia, and less then 1% to Belarus.

Many Non-Governmental Organizations have promptly responded to the humanitarian emergency by gathering funds, medicines, food, clothes, and essential goods, and by sending their staff on the field to provide support and assistance to people in needs. On the 4th of March, the EU has responded with the activation of the Temporary Protection Directive.^5^

Besides defining the decision-making procedure needed to trigger, extend, or end temporary protection, the Directive lists the rights for the beneficiaries of temporary protection. Among these rights, there is the access to employment, suitable accommodation, social welfare, medical care, and education for people under 18.

Member States need to be ready to host hundreds of thousands of people avoiding them to fall into a limbo made of administrative delays and logistic unpreparedness. Moreover, the European Commission is looking for a fair way to financially support each Member State in its effort to welcome and accommodate people fleeing Ukraine. Therefore, it is crucial to assess the number of people reaching each of the EU countries and regions.

Unfortunately, the UNHCR warns that *“data of arrivals in Schengen countries (Hungary, Poland, Slovakia) bordering Ukraine only represents border crossings into that country, but we estimate that a large number of people have moved onwards to other countries”*. In other words, it provides estimates on the number of border-crossings from Ukraine to the neighboring countries, rather than on the number of people hosted by each country. To date, there are no reliable data on the actual number of people who have left Ukraine and reached the EU countries. This is where non-traditional data sources can help filling a gap by providing such estimates on a (near-) real time basis.

## Facebook advertising data

This section describes data and methodology used for the analysis. Facebook’s advertising platform provides anonymous and aggregated information on Facebook users through a dedicated Application Programming Interface (API) [26]. This platform enables advertisers to run advertisements targeted at users of Facebook’s family of applications and services, which include Facebook, Instagram, Messenger, and the Audience Network. It can be used to retrieve the estimates of the Monthly Active Users who are eligible to be shown an advertisement given a set of user characteristics. MAUs include users active in the previous 30 days. In this work, we focus on two main user characteristics, namely the country of residence and the language of the users. This latter attribute is provided by Facebook advertising platform to target people with language other than common language for a location. Since Facebook does not directly provide information on the nationality of its users, we use the language as a proxy to infer users of Ukrainian nationality. To test this hypothesis, in Sect. 5 we compare the Ukrainian-speaking MAUs relative to the month before the Russian invasion of Ukraine with official Ukrainian diaspora data in the EU from EUROSTAT.^6^ Our implicit assumption is that the number of Facebook MAUs relative to Ukrainian-speaking users in each EU country is fairly stable before the war and it is therefore comparable with the latest diaspora records.

However, using the language option has a drawback, since starting from the 23th of August 2021 target advertisement to people under the age of 18 is not available.^7^ To take this into consideration, during the comparison with diaspora data we only consider the Ukrainian citizens over 18.

It is worth highlighting that self-declared Ukrainian-speaking MAUs do not reflect the total Ukrainian population, the two main reasons being (i) not all Ukrainians use Facebook (in particular, under 13 people cannot open an account); (ii) Ukrainian is the language spoken by the vast majority of people in the country, but other languages are also common, in particular Russian.^8^ Nevertheless, Ukrainian language is not very diffuse outside Ukraine and the neighboring countries, and its diffusion in Europe is very limited.^9^ Moreover, we acknowledge that not all people fleeing Ukraine are Ukrainian nationals. In fact, the EU Temporary Protection Directive is directed to everyone fleeing the country, regardless of their nationality.

Recent studies have focused on the reliability of the socio-demographic information provided by the Facebook’s advertising platform [26, 28–30]. Sances [29] and Grow et al. [28] observe that the information reported by the users upon creating their account, in particular those that are less likely to change over time (*e.g.* gender, age), are generally accurate and more reliable than other information which are inferred by Facebook advertising algorithms, such as the region of residence. Grow et al. [28] report that misclassifications between the actual characteristics of the users and the ones provided by Facebook are most likely to occur for the region of residence, which is partially inferred by Facebook and may change frequently, thereby increasing the chance for erroneous classifications. However, Sances [29] states that classifications on the region of residence are more likely to be correct in larger regions than in smaller regions. Since we are looking at changes in MAUs at national scale, we assume the considered geographical resolution to be sufficiently large to neglect major classification errors.

It is important to point out that Facebook estimates are not designed to match population, census estimates, or other sources, and are not to be considered as a proxy for monthly or daily active users on Meta, or engagement.^10^ They may differ depending on factors such as:

- how many Facebook apps and services accounts a person has.
- how many temporary visitors are in a particular geographic location at a given time.
- Facebook user-reported demographics.

However, recent studies indicate that despite measurement issues and selection bias, it is potentially feasible to derive robust estimates of demographic indicators from tabulations of Facebook users [25, 26, 31, 32]. The same works present approaches to generate bias-adjusted population estimates and demographic counts to derive the actual distributions for specific audiences of interest. Similarly to [31], we estimate the Facebook penetration rate in Ukraine by dividing the prewar Ukrainian-speaking MAUs in Ukraine by the population over 18 in Ukraine provided by the Ukrainian statistical office.^11^ In Sect. 5 we explain in detail how this is calculated and how we use the estimated penetration rate as a correction factor for Facebook audience estimates in each country.

One key aspect when using non-traditional data is validating them with reliable sources when available. To this date, public data on the actual flows of people fleeing Ukraine are very limited. We rely on data on refugee influx from Ukraine in neighboring countries available at the Operational Data Portal of UNHCR. In Sect. 5 we compare the weekly change in Facebook MAUs with daily UNHCR inflow data for the five weeks following the beginning of the war. The comparison is made for the EU countries neighboring Ukraine.

MAU estimates refer to a 30 days time span, and we don’t know if the target audience for a given country might be inflated by users transiting in a country to reach another country of destination; for instance, a user travelling in different countries will be counted as many times as the number of countries where he or she has interacted with Facebook applications. As a consequence, when looking at the increase in MAUs through time it may not be possible to discern how much of the change is to be attributed to Ukrainians merely transiting the country and how much to Ukrainians actually settling in. For the same reason, insights on outflows may not be immediately visible, as the effect on the multiple counts would take some time to fade out. However, to the best of the author’s knowledge it is not clear if the estimation of the target audience provided by the Facebook’s advertising platform are already corrected for this bias or not.

UNHCR data also have some caveats. First, they represent the arrivals (*i.e.* inflow) of people fleeing Ukraine towards neighboring countries, not the actual number of people displaced in a country at a given time. Second, the right to move freely within the Schengen area means there are very few border controls. The data of arrivals in Schengen countries (Hungary, Poland, Slovakia) bordering Ukraine therefore only represents border crossings into that country, but UNHCR estimates that a large number of people have moved onwards to other countries. Nevertheless, these figures represent the only tried and tested publicly available information as of the time of writing, and we compare them with our data to check if we find a similarity in the trends.

### Data collection

An automated script developed at the JRC’s Knowledge Centre on Migration and Demography (KCMD)^12^ collects data on a weekly basis by making requests to the Facebook’s Marketing Application Programming Interface. The same script has already been employed in Spyratos et al. [31]. Using the API it is not possible to query historical data. For this reason, every time the script runs it stores the response of each query in a database, allowing us to have a time series of the data.

Our script makes requests to the Marketing API to retrieve data on the estimated number of people that satisfy a set of characteristics, as described in the documentation at Meta for Developers website.^13^ For this study, these are the country of residence and the language of the users, which can be requested by setting the proper parameters under the targeting_spec field of the delivery_estimate endpoint.^14^

An example of a query looks like the following: 
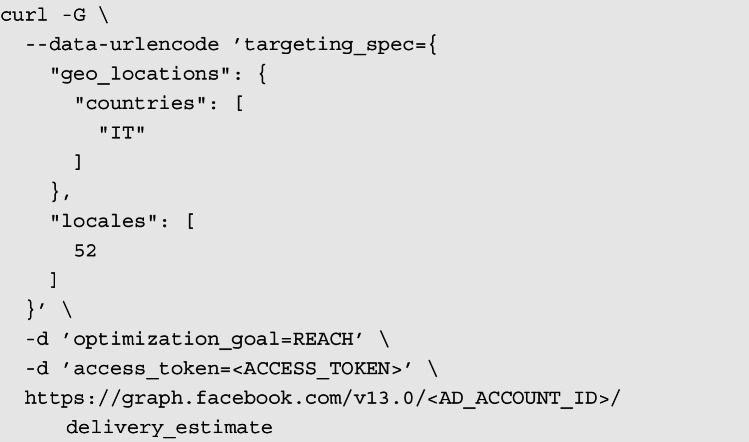


In the above example, we request an audience estimate of users based in Italy and who speak Ukrainian. By setting the optimizationz_goal field to REACH, we ensure the ad set is optimized to reach the most unique users of each day. In other words, this is set to serve the maximum number of people. The locales field allows to specify the language of the user. Here, 52 corresponds to the “Ukrainian” language.^15^

The response of the API looks like the following (only the interesting data are shown): 
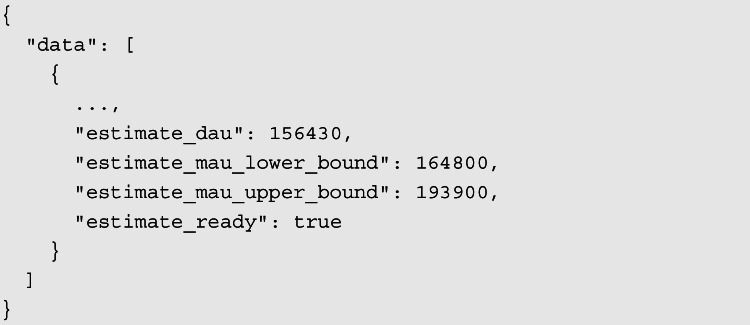


The result refers to the time the query was sent (estimate_dau) and the 30 days prior to it in the case of the estimate_mau* fields. estimate_mau_lower_bound and estimate_mau_upper_bound represent the lower and upper bounds of the estimated number of people that have been active on the selected platforms and satisfy the targeting spec in the past month respectively.^16^ By not restricting the data collection to users of one or more specific target application such as Facebook or Instagram, we are implicitly selecting all possible Facebook’s platforms and services, thus covering the largest possible number of users that meet our chosen criteria.

Finally, the value of MAUs we use throughout this work is the average between the lower bound and the upper bound estimates coming from the API.

## Estimating refugee flows at national level

Since Facebook does not provide information on the nationality of its users, we use the attribute language declared by the users as a proxy to infer users of Ukrainian nationality. To test this hypothesis, we analyse the correlation between the Ukrainian-speaking Facebook MAUs relative to 30 days prior the Russian invasion of Ukraine and official Ukrainian diaspora in EU at national level provided by EUROSTAT.

Similarly to [31], we estimate the Facebook penetration rate of Ukrainian speakers in Ukraine by dividing the prewar Ukrainian-speaking MAUs estimate in Ukraine by the population over 18 in Ukraine provided by the Ukrainian statistical office (see Eq. (1)). We then use the result of this estimation as a correction factor for Facebook audience estimates in each country (see Eq. (2)). By doing so, we up-adjust the estimates correcting for the fact that not all the Ukrainians that have left the country are on Facebook.

1 $$\begin{aligned}& \mathrm{FB}\mathit{pr}_{\mathit{UA},\geq 18\mathit{yo}} = \mathrm{MAU}_{\mathit{UA},w0} \:/\:\mathrm{POP}_{\mathit{UA}, \geq 18\mathit{yo}}, \end{aligned}$$

2 $$\begin{aligned}& \mathrm{MAU}\mathit{adj}_{c} = \mathrm{MAU}_{c}\:/\:\mathrm{FB}\mathit{pr}_{\mathit{UA},\geq 18\mathit{yo}}. \end{aligned}$$

In Eq. (1), $\mathrm{FB}\mathit{pr}_{\mathit{UA},\geq 18\mathit{yo}}$ is the Facebook Penetration rate of Ukrainian speakers in Ukraine over 18, $w_{0}$ represents the first available week (*i.e.* prewar, relative to data collected the 25th February and referring to 30 days prior to it), and UA refers to the ISO 3166-1 alpha-2 country code of Ukraine.^17^ In Eq. (2), $\mathrm{MAU}_{c}$ and $\mathrm{MAU}\mathit{adj}_{c}$ are respectively the original and the up-adjusted MAUs in the hosting country *c*.

**Figure 1:**
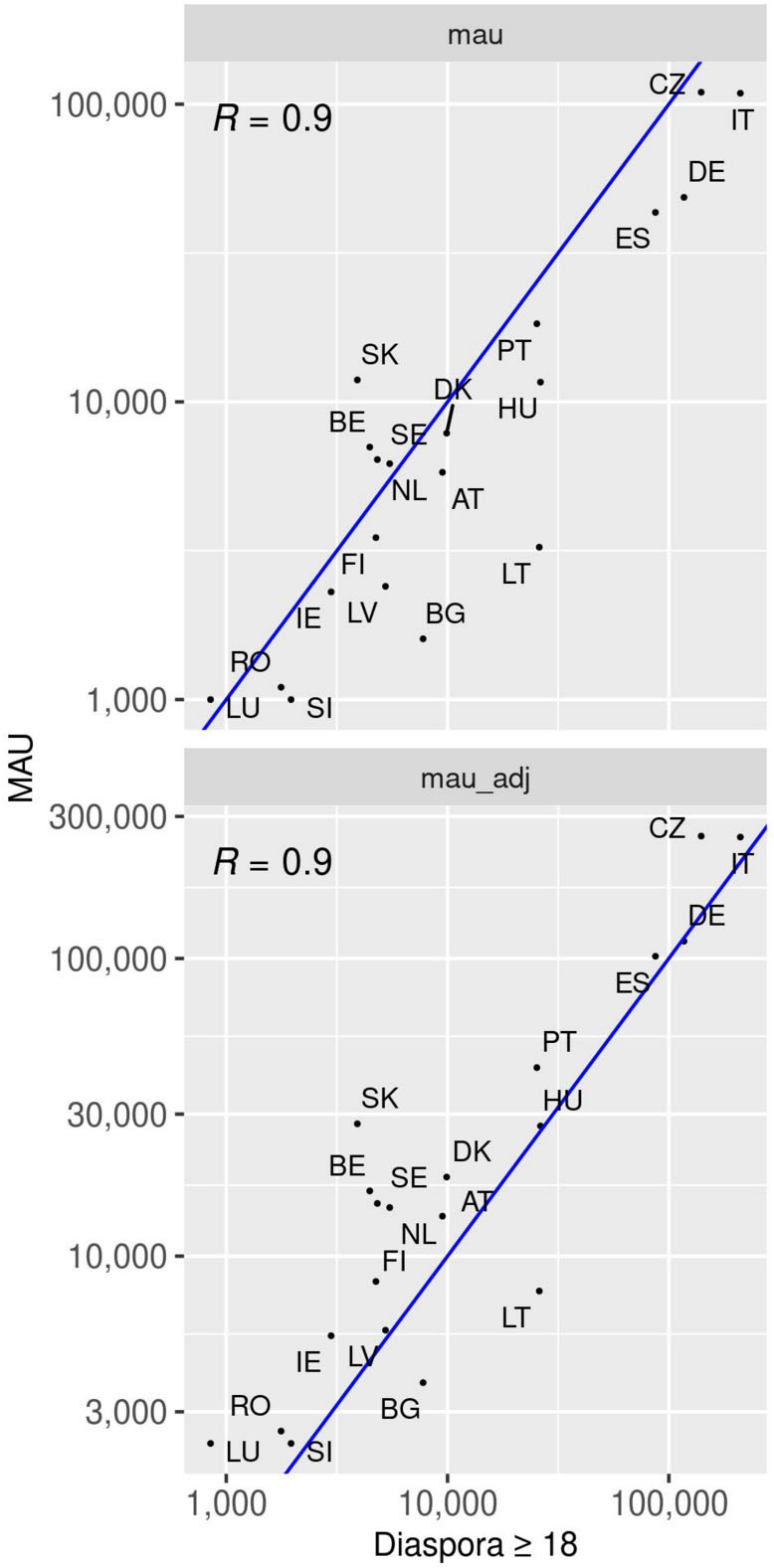
*Prewar MAUs vs over 18 Ukrainian diaspora*. Scatter-plot (logarithmic scale) of prewar Facebook MAUs at national level against Ukrainian diaspora in 21 EU countries (official statistics relative to 2021). Upper plot: original MAUs. Lower plot: adjusted MAUs

Figure 1 shows the scatter-plots of the Ukrainian diaspora in most of the EU countries and Ukrainian-speaking MAUs (both original and adjusted with the penetration rates) at national level relative to the month before the Russian invasion of Ukraine.

In both cases, we observe a high correlation (Pearson’s $r=0.9$, $p<0.0001$) with official diaspora data for both the original (lower-bound), and the adjusted (upper-bound) MAUs. Therefore, the assumption to use Ukrainian language attribute from Facebook as a proxy of nationality seems to be reasonable (at least for the Ukrainians).

We then inspect the variation in the number of Ukrainian-speaking Facebook MAUs between the first prewar set of data and the last available period. We use the results as a proxy for the increase (decrease) of the number of Ukrainians within each EU country.

We estimate the percentage share of increase (decrease) in Ukrainian stocks among the 27 EU Member States in the last available week for each country *c* as:

3 $$ \Delta \mathrm{UA}_{w_{5},c}\% = \frac{\Delta \mathrm{UA}_{w_{5},c}}{\sum_{i = 1}^{27} \Delta \mathrm{UA}_{w_{5},c_{i}}}, $$

where $\Delta \mathrm{UA}_{w_{5}}$ is defined as:

4 $$ \Delta \mathrm{UA}_{w_{5}} = \mathrm{UA}\mathit{stock} \cdot{ \frac{\mathrm{MAU}_{w_{5}}-\mathrm{MAU}_{w0}}{\mathrm{MAU}_{w_{0}}}}. $$

In the above equations, $w_{0}$ and $w_{5}$ represent the baseline (*i.e.* prewar) week, and the last available week respectively, and UA*stock* is the Ukrainian population provided by EUROSTAT.

**Figure 2:**
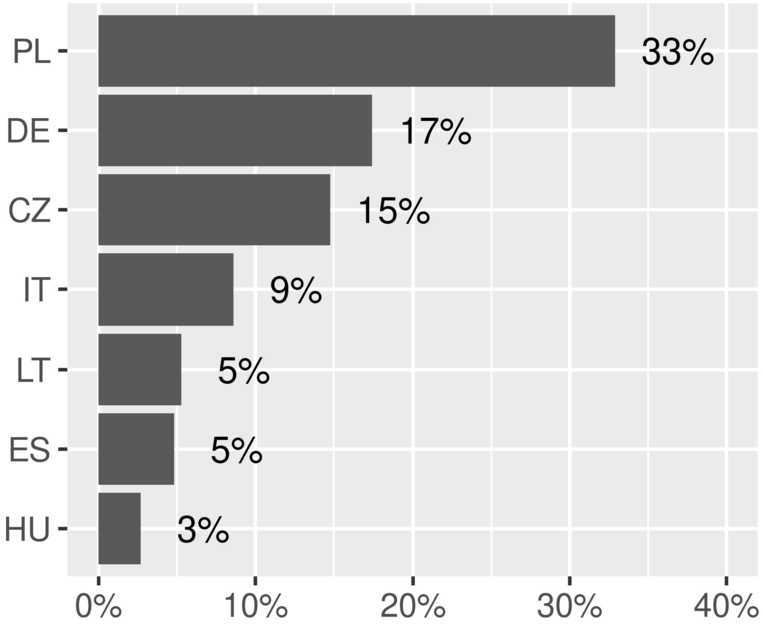
*Percentage share of Ukrainian stocks in EU at week 5*. Percentage share of estimated Ukrainian stocks change in the EU countries between the beginning of the war and the fifth week. Only countries with significant increments ($>2\%$) are shown

We find that $\Delta \mathrm{UA}_{w_{5},c}\%$ is positive for all the EU Member States. Figure 2 shows the increase for the countries where we observe a significant change ($>2\%$).

In our analysis, Poland shows the greatest increment of percentage share of estimated Ukrainian stocks, accounting for 33% of the total share in the EU. This is in line with the UNHCR data, where Poland is reported as the country with the highest “inflow of refugees from Ukraine” as of the 4th of April 2022. The absence of Slovakia and Romania—two of the four EU countries bordering Ukraine—might indicate that despite the proximity to Ukraine, they appear to be more transiting countries for refugees rather than a place where they settle, since their percentage share of Ukrainian stocks is low compared to other Member States. On the other hand, the presence of farther countries with a high percentage share such as Germany and to a lesser degree Italy might indicate that these countries are potentially a final destination.

**Figure 3:**
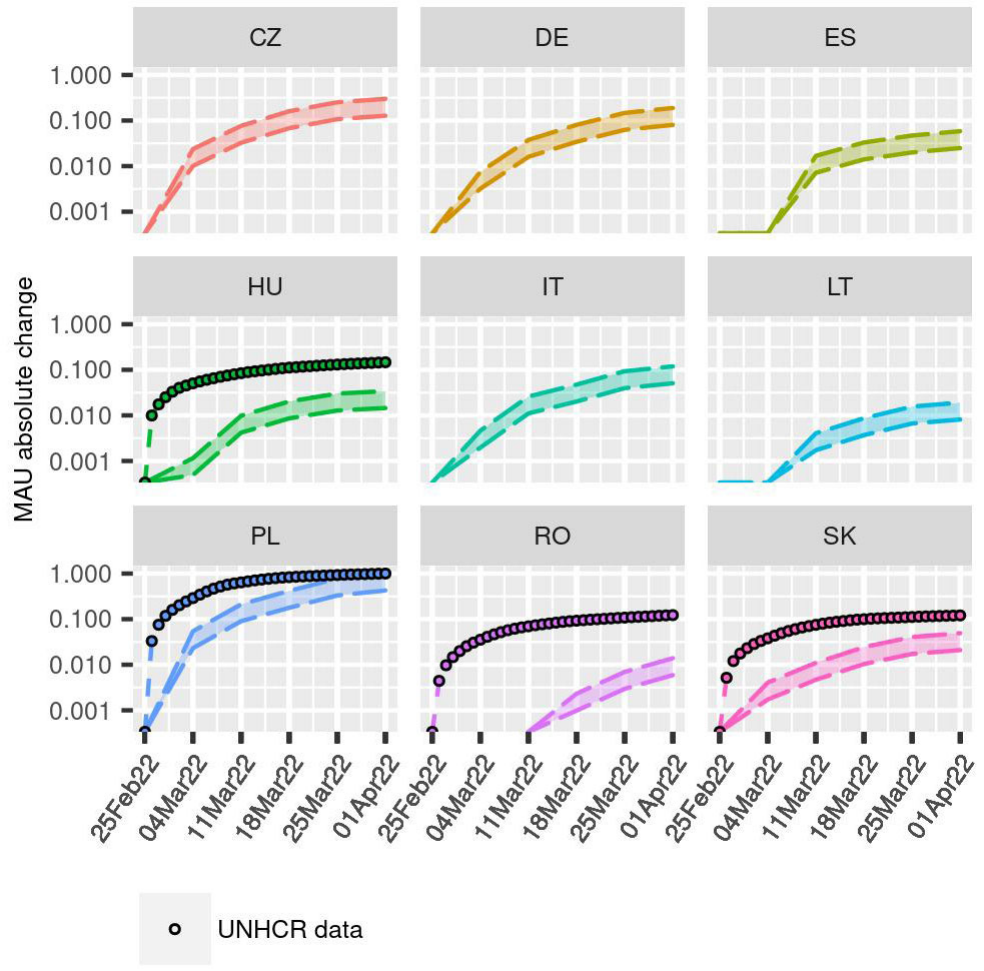
*Facebook MAUs vs UNHCR data*. Normalized weekly absolute change of Facebook Monthly Active Users and normalized daily absolute change of arrivals from UNHCR for the available weeks (logarithmic scale)

Our final analysis focuses on the weekly increase of Ukrainian-speaking Facebook MAUs in the EU Member States. Figure 3 shows the normalized absolute change of MAUs for the five weeks following the start of the war. The countries shown here are the same of Fig. 2, plus Slovakia and Romania (the remaining EU countries neighboring Ukraine). The boundaries of the ribbons in the figure represent the normalized original MAU (lower bounds), and normalized adjusted MAUs (upper bound).

In all instances but one (Romania), the increase seems steeper in the first weeks. Poland is the country with the greatest increase of MAUs overall, followed by Czechia, and Germany. Each segment represents the increase of MAUs speaking Ukrainian with respect to the previous period. We do not observe any decrease in these countries. Romania does not show any significant increase of Ukrainian-speaking Facebook MAUs until week 2. It is hard to say whether this has to be attributed to an issue in the calculation of the audience estimate by the Facebook advertising platform, or if the Ukrainian-speaking Facebook MAUs were actually stable in this period.

It is interesting to focus on the change in the slope between subsequent segments: in the future and if the situation will reach a new stationary point, we might expect this slope to decrease in the transiting countries, and increase in the final destinations.

In the same figure, we show the normalized absolute change of the cumulative daily arrivals to the EU countries neighboring Ukraine, as reported by UNHCR. The normalization of the Facebook and UNHCR data is done to ensure the comparability of the trends, and it is calculated as:

5 $$ v^{\prime }_{i} = v_{i}\:/ \max{(V)}. $$

In Eq. (5), $v^{\prime }_{i}$ and $v_{i}$ are respectively the normalized and the original *i*th value of either Facebook MAUs or UNHCR dataset; $\max{(V)}$ is the maximum value of the series, with $V = \{v_{1},\dots ,v_{N}\}$ and *N* being the number of total observations in the series. The original Facebook MAUs values are normalized using the maximum of the up-adjusted MAUs series to preserve the lower-bound.

From the results of our analysis, it seems that UNHCR data and Facebook MAUs have similar trends, except in Romania. In this case, it is not clear if this is due to an issue in the calculation of the audience estimate, an actual stability of the number of Ukrainian-speaking Facebook users in Romania, or other reasons. It would be useful to focus on this country to see how future observations will compare to UNHCR data in the following weeks. Moreover, we observe a gap between Facebook estimates and UNHCR data in the first week in Hungary, with UNHCR reporting a larger increase. The gap is then reduced in the second week by an increase in MAUs, with the rest of the period showing very similar slopes. This would indicate that overall both data have a similar trend, although the Facebook estimates seem to present a delay in capturing it compared to UNHCR data.

## Discussion and conclusions

In this work, we analyse Ukrainian-speaking Facebook MAUs to monitor the flows of people fleeing Ukraine towards the European Union after the Russian invasion. Our results show that these non-traditional data can provide fast and preliminary insights on the effects of the ongoing Ukrainian crisis on the international migratory flows.

We find a strong correlation (Pearson’s $r=0.9$, $p<0.0001$) between the Ukrainian-speaking MAUs in the first available week (prewar) and the official statistics of the over 18 Ukrainian diaspora in the EU countries. This supports the assumption that the language attribute reported by the users can be considered a good proxy to infer the nationality of Ukrainian users, an information which is not provided by the Facebook advertising platform.

In order to estimate the flows of Ukrainians in the EU Member States following the military aggression of Ukraine, we compute the variation in the number of estimated Ukrainian stocks between the beginning of the war and the last available week in our dataset (the fifth week). We observe an increase in all the EU countries, with Poland registering the highest percentage share (33% of the overall increase), followed by Germany (17%), and Czechia (15%). Interestingly, Slovakia and Romania, both EU countries neighboring Ukraine, present low percentage share ($<2\%$), most likely indicating that despite the proximity to Ukraine, they appear to be more transiting countries for refugees rather than a place where they settle. On the other hand, farther countries with a high percentage share such as Germany and to a lesser degree Italy are more likely to represent potential final destinations.

By looking at the weekly absolute change of MAUs in the five weeks period following the start of the war, we show an increasing trend in all EU Member States, with Poland being the country with the greatest increase in absolute terms, followed by Czechia, and Germany. We observe a steeper increase in Facebook MAUs in all countries in the first weeks, especially in those close to Ukraine (except Romania). This might indicate that some of the users who fled Ukraine towards the EU did not settled in the first country they reached, but most probably moved on towards farther destinations. Finally, we compare the increment curve in the countries neighboring Ukraine with data on refugees from Ukraine from the UNHCR, and we observe that they present very similar trends except for Romania. Our future research will focus on this country to understand if the trend seen in the UNHCR data is captured with more observations even with a delay or not.

It should be taken into account that it is not clear if MAUs estimates provided by the Facebook advertising platform are corrected for the potential bias of multiple counts. If this is not the case, since they cover a 30 days period, data collected during the first five weeks of the war would not allow identifying possible secondary movements of people who fled the country. Depending on the magnitude of these secondary movements, there may be an overestimation of the outflows. The activity of an Ukrainian-speaking Facebook user moving from the first receiving country to another country within the 30 days would be counted multiple times, one for each country where the user activity was recorded. As a consequence, it would not possible to discern how much of the increase in the number of Ukrainians in a country could be attributed to refugees merely transiting the country and how much to refugees actually settling in. Additional collections of data in different time intervals would allow to estimate the extent of secondary movements. However, with the present data and in the case a correction for multiple counts is missing, we can assume that the values in the EU Member States that are not bordering with Ukraine would be less biased because they more likely represent a final destination due to their distance from the origin.

Some caveats to this study should be highlighted. The first is related to the representativeness of the Facebook’s data on Ukrainian population. We estimate the Facebook penetration rate of Ukrainian speakers in Ukraine to be around 43%. This estimation is based on the number of Ukrainian-speaking MAUs in Ukraine in prewar times over the Ukrainian population above 18 years, as reported by the Ukrainian statistical office. The reason we are not considering the population below 18 years of age in the calculation is because it is not available in the audience estimate when using the language attribute.

Second, since our sample is restricted only to Facebook users who speak Ukrainian, we exclude from the analysis people fleeing the country who speak other languages. The reason behind this choice of sample restriction is the impossibility to distinguish, in the receiving countries, between Facebook users that fled Ukraine and speak, for example, Russian (the second most common language in Ukraine), and the Facebook users moving from other Russian-speaking countries. However, Ukrainian is the language spoken by the vast majority of people in Ukraine, and its diffusion in Europe is very limited.

Due to the imposition of the martial law in Ukraine on the 24th of February 2022,^18^ men between 18 and 60 years are not allowed to leave the country. Therefore most of the population that has fled Ukraine should be females, minors, and the elderly. A recent report on social media diffusion in Ukraine^19^ shows that most Ukrainian Facebook users are females (60.4%), and users interact with Facebook mainly via their phones (95.6%). For these reasons, even though we cannot capture the under 18, since a big portion of the target population should be female, we might assume that the actual Facebook penetration of our target audience under these circumstances is higher than in normal conditions, and therefore the insights deriving from Facebook MAUs should be more representative of the displaced persons. Moreover, since it is very likely that they are carrying a phone with them (to communicate with their parents and friends and for other reasons), they still would have the possibility to interact with the Facebook application should they need to.

Despite the above-mentioned caveats, and in the absence of official data on the number of people displaced outside Ukraine, our study shows that data derived from social media could offer timely insights on international mobility during crises. These data could support initiatives aimed at providing humanitarian assistance to the displaced people, as well as local and national authorities to better manage their reception and integration. Finally, the same data could allow monitoring international flows of people where this information is often missing, due to the freedom to move across countries without strict border control, as in the Schengen area.

## Data Availability

All data used in this study are openly available. Recent Meta data, describing the same attributes as the one used in the study, are openly available from Meta, through Facebook’s Marketing Application Programming Interface (https://developers.facebook.com/docs/marketing-apis/). We confirm that we, as authors, did not have any special data access privileges that others would not have. Due to legal requirements regarding the publication of Meta data, the minimal data underlying the results of this study are available for academic purposes upon request. The code to reproduce the results in this manuscript is available at https://code.europa.eu/kcmd-datainno/fb-adv4UKR. Data can be requested from the corresponding author Umberto Minora (umberto.minora@ec.europa.eu), European Commission—Joint Research Centre (JRC) Demography, Migration and Governance Unit, TP 266, Via E. Fermi 2749, 21027 Ispra (VA), Italy.

## Footnotes

1 (EU)2021/836

2 Throughout the paper, we refer to Monthly Active Users taking into account this definition.

3 Global Protection Cluster IDP-Figures-in-Ukraine-18-March.pdf and IOM’s Ukraine Internal Displacement Report IOM_IDP_Estimates_UKR_16MAR2022_Round_1_full_report_v2.pdf. Last accessed the 4th of April 2022.

4 UNHCR Operational data Portal—Ukraine https://data2.unhcr.org/en/situations/ukraine. Last accessed the 4th of April 2022.

5 COUNCIL IMPLEMENTING DECISION (EU) 2022/382 of 4 March 2022 https://eur-lex.europa.eu/legal-content/EN/TXT/?uri=uriserv%3AOJ.L_.2022.071.01.0001.01.ENG&toc=OJ%3AL%3A2022%3A071%3ATOC. Last accessed the 4th of April 2022.

6 https://appsso.eurostat.ec.europa.eu/nui/show.do?dataset=migr_pop1ctz

7 https://www.facebook.com/business/help/229435355723442

8 According to the 2001 Ukrainian census, Ukrainian and Russian are respectively the mother tongue of 67.5% and 29.6% of the population. http://2001.ukrcensus.gov.ua/eng/results/general/language/

9 It is estimated that 91% of native speakers of Ukrainian live in Ukraine [27].

10 https://www.facebook.com/business/help/1665333080167380?id=176276233019487. Last accessed the 4th of April 2022.

11 Data from http://www.ukrstat.gov.ua/. Last accessed the 7th of September 2022.

12 https://knowledge4policy.ec.europa.eu/migration-demography_en

13 https://developers.facebook.com/docs/marketing-apis

14 https://developers.facebook.com/docs/marketing-api/reference/ad-campaign/delivery_estimate/

15 For a list of possible languages and their locale codes, see https://developers.facebook.com/docs/marketing-api/audiences/reference/targeting-search#locale

16 https://developers.facebook.com/docs/marketing-api/reference/ad-campaign-delivery-estimate

17 https://www.iso.org/iso-3166-country-codes.html. Last accessed the 16th of May 2022.

18 https://www.president.gov.ua/en/news/prezident-pidpisav-ukaz-pro-zaprovadzhennya-voyennogo-stanu-73109. Last accessed the 9th April 2022

19 DataReportal https://datareportal.com/reports/digital-2020-ukraine?rq=ukraine. Last accessed the 20th May 2020.

## References

[1] Bosco C, Grubanov-Boskovic S, Iacus SM, Minora U, Sermi F, Spyratos S (2022) Data innovation in demography, migration and human mobility (EUR 29333 EN). 10.2760/958409

[2] Bengtsson L, Lu X, Thorson A, Garfield R, Von Schreeb J (2011). Improved response to disasters and outbreaks by tracking population movements with mobile phone network data: a post-earthquake geospatial study in Haiti. PLoS Med.

[3] Lu X, Bengtsson L, Holme P (2012). Predictability of population displacement after the 2010 Haiti earthquake. Proc Natl Acad Sci.

[4] Wilson R, Zu Erbach-Schoenberg E, Albert M, Power D, Tudge S, Gonzalez M, Guthrie S, Chamberlain H, Brooks C, Hughes C, Pitonakova L, Buckee C, Lu X, Wetter E, Tatem A, Bengtsson L (2016) Rapid and near real-time assessments of population displacement using mobile phone data following disasters: the 2015 Nepal earthquake. PLoS Curr 8. 10.1371/currents.dis.d073fbece328e4c39087bc086d694b5c 10.1371/currents.dis.d073fbece328e4c39087bc086d694b5c PMC4779046 26981327

[5] Li T, Dejby J, Albert M, Bengtsson L, Lefebvre V (2019) Detecting individual internal displacements following a sudden-onset disaster using time series analysis of call detail records. arXiv preprint. arXiv:1908.02377

[6] Flowminder Foundation (2021) Flowminder Foundation: population movements following the haiti earthquake on 14 august 2021 and the tropical depression grace, estimated with mobile operator data from digicel haiti: report from 27 August. Technical report. https://www.flowminder.org/media/qtsdp1ty/haitiearthquake_report_27-aug_report-2_eng.pdf

[7] Isaacman S, Frias-Martinez V, Frias-Martinez E (2018). Modeling human migration patterns during drought conditions in La Guajira, Colombia. Proceedings of the 1st ACM SIGCAS conference on computing and sustainable societies. COMPASS ’18.

[8] Lu X, Wrathall DJ, Sundsøy PR, Nadiruzzaman M, Wetter E, Iqbal A, Qureshi T, Tatem A, Canright G, Engø-Monsen K, Bengtsson L (2016). Unveiling hidden migration and mobility patterns in climate stressed regions: a longitudinal study of six million anonymous mobile phone users in Bangladesh. Glob Environ Change.

[9] Rayer S (2018) Estimating the migration of Puerto Ricans to Florida using flight passenger data. Bureau of Economic and Business Research, University of Florida

[10] Jia S, Kim SH, Nghiem SV, Doherty P, Kafatos MC (2020). Patterns of population displacement during mega-fires in California detected using Facebook disaster maps. Environ Res Lett.

[11] Li T, Bowers R, Seidu O, Akoto-Bamfo G, Bessah D, Owusu V, Smeets L (2021). Analysis of call detail records to inform the COVID-19 response in Ghana—opportunities and challenges. Data Policy.

[12] Wesolowski A, Buckee CO, Bengtsson L, Wetter E, Lu X, Tatem AJ (2014) Commentary: containing the ebola outbreak-the potential and challenge of mobile network data. PLoS Curr 6 10.1371/currents.outbreaks.0177e7fcf52217b8b634376e2f3efc5e PMC4205120 25642369

[13] Cot C, Cacciapaglia G, Sannino F (2021). Mining Google and Apple mobility data: temporal anatomy for COVID-19 social distancing. Sci Rep.

[14] Snoeijer BT, Burger M, Sun S, Dobson RJB, Folarin AA (2021). Measuring the effect of non-pharmaceutical interventions (NPIs) on mobility during the COVID-19 pandemic using global mobility data. npj Digit Med.

[15] Yilmazkuday H (2021). Stay-at-home works to fight against COVID-19: international evidence from Google mobility data. J Hum Behav Soc Environ.

[16] Hu T, Guan WW, Zhu X, Shao Y, Liu L, Du J, Liu H, Zhou H, Wang J, She B, Zhang L, Li Z, Wang P, Tang Y, Hou R, Li Y, Sha D, Yang Y, Lewis B, Kakkar D, Bao S (2020). Building an open resources repository for COVID-19 research. Data Inf Manag.

[17] Lai S, Bogoch II, Ruktanonchai NW, Watts A, Lu X, Yang W, Yu H, Khan K, Tatem AJ (2020) Assessing spread risk of Wuhan novel coronavirus within and beyond China, January–April 2020: a travel network-based modelling study. medRxiv. 10.1101/2020.02.04.20020479

[18] Carammia M, Iacus SM, Wilkin T (2022) Forecasting asylum-related migration flows with machine learning and data at scale. Nature Scientific Reports. Preprint. https://arxiv.org/abs/2011.04348 10.1038/s41598-022-05241-8 PMC8795256 35087096

[19] Suleimenova D, Bell D, Groen D (2017). A generalized simulation development approach for predicting refugee destinations. Sci Rep.

[20] Corbane C, Kemper T, Freire S, Louvrier C, Pesaresi M (2016) Monitoring the Syrian Humanitarian Crisis with the JRC’s Global Human Settlement Layer and Night-Time Satellite Data vol. LB-NA-27933-EN-C (print), LB-NA-27933-EN-N (online). Publications Office of the European Union, Luxembourg (Luxembourg). 10.2788/48956 (print), 10.2788/48956 (online)

[21] Bharti N, Lu X, Bengtsson L, Wetter E, Tatem AJ (2015). Remotely measuring populations during a crisis by overlaying two data sources. Int Health.

[22] Curry T, Croitoru A, Crooks A, Stefanidis A (2019). Exodus 2.0: crowdsourcing geographical and social trails of mass migration. J Geogr Syst.

[23] Mazzoli M, Diechtiareff B, Tugores A, Wives W, Adler N, Colet P, Ramasco JJ (2020). Migrant mobility flows characterized with digital data. PLoS ONE.

[24] Hausmann R, Hinz J, Yildirim MA (2018) Measuring Venezuelan emigration with Twitter. Kiel Working Paper 2106, Kiel. http://hdl.handle.net/10419/179127

[25] Palotti J, Adler N, Morales-Guzman A, Villaveces J, Sekara V, Garcia Herranz M, Al-Asad M, Weber I (2020). Monitoring of the venezuelan exodus through Facebook’s advertising platform. PLoS ONE.

[26] Zagheni E, Weber I, Gummadi K (2017). Leveraging Facebook’s advertising platform to monitor stocks of migrants. Popul Dev Rev.

[27] Lewis MP (2009). Ethnologue: languages of the world.

[28] Grow A, Perrotta D, Del Fava E, Cimentada J, Rampazzo F, Gil-Clavel S, Zagheni E, Flores RD, Ventura I, Weber I et al (2021) How reliable is Facebook’s advertising data for use in social science research? Insights from a cross-national online survey. Technical report, Max Planck Institute for Demographic Research, Rostock, Germany

[29] Sances MW (2021). Missing the target? Using surveys to validate social media ad targeting. Polit Sci Res Methods.

[30] Pötzschke S, Braun M (2017). Migrant sampling using Facebook advertisements: a case study of Polish migrants in four European countries. Soc Sci Comput Rev.

[31] Spyratos S, Vespe M, Natale F, Weber I, Zagheni E, Rango M (2019). Quantifying international human mobility patterns using Facebook network data. PLoS ONE.

[32] Ribeiro FN, Benevenuto F, Zagheni E (2020). How biased is the population of Facebook users? Comparing the demographics of Facebook users with census data to generate correction factors. 12th ACM conference on web science. WebSci ’20.

